# Validated CFD experimental study of heat transfer in water bath heaters at city gate stations

**DOI:** 10.1016/j.isci.2026.116465

**Published:** 2026-06-26

**Authors:** Hamidreza Mortazavy Beni, Seyed Hossein Sajadian, Afshin Ahmadi Nadooshan, Hamed Mortazavi

**Affiliations:** 1 Department of Biomedical Engineering, Ars.C., Islamic Azad University, Arsanjan, Iran; 2 National Iranian Gas Company (NIGC), Fars Province, Shiraz, Iran; 3 Department of Mechanical Engineering, Shahrekord University, Shahrekord, Iran; 4 Sefid Dasht Steel Complex, Shahrekord, Chaharmahal and Bakhtiari Province, Iran

**Keywords:** Energy engineering, Mechanical engineering, Thermofluids

## Abstract

This study develops and validates a conjugate computational fluid dynamics (CFDs) framework to quantify heat-transfer paths in indirect water-bath heaters used at city-gate stations and to assess the influence of atmospheric-burner settings. A full 3-D model of a station heater—resolving the fire-tube, water bath, and process-gas coil—was built and solved with RANS turbulence and coupled solid-fluid heat transfer at two representative operating loads (20,000 and 40,000 m^3^ h^−1^). Grid-independence was demonstrated (change in coil-outlet temperature <0.5% beyond ∼1.67 M cells), and integral energy balances closed within the prescribed tolerances. The validated solution shows a buoyancy-driven bath with a warm cap beneath the top head and a cooler descending core through the coil bundle (mean bath ≈48°C; characteristic bath speeds ≤0.05 m s^−1^). In the coil, the gas warms monotonically from ∼10°C to the station setpoint (∼35°C–36°C), with most duty delivered on the first pass; local velocity peaks (∼6 m s^−1^) occur at elbow inner radii. On the fire side, the premixed jet forms an upward-lifting hot core (peak ≈880°C), producing a top-wall hot streak that concentrates heat flux but elevates wall metal temperature. Analysis indicates that high excess-air operation—typical of atmospheric burners—penalizes efficiency mainly through stack sensible losses rather than bath-side limitations. The model highlights practical levers for improvement—moderating excess air and tuning the primary/secondary split, gentle bath recirculation or baffling, and coil-pass placement—which can be screened numerically before field implementation.

## Highlights

• A validated CFD model reproduces the thermal behavior of industrial heaters
• Natural convection generates persistent thermal stratification in the bath
• Buoyancy induces an upper wall hot streak inside the fire tube
• Simple operational adjustments can enhance the heater's thermal performance

## Graphical abstract

**Figure:**
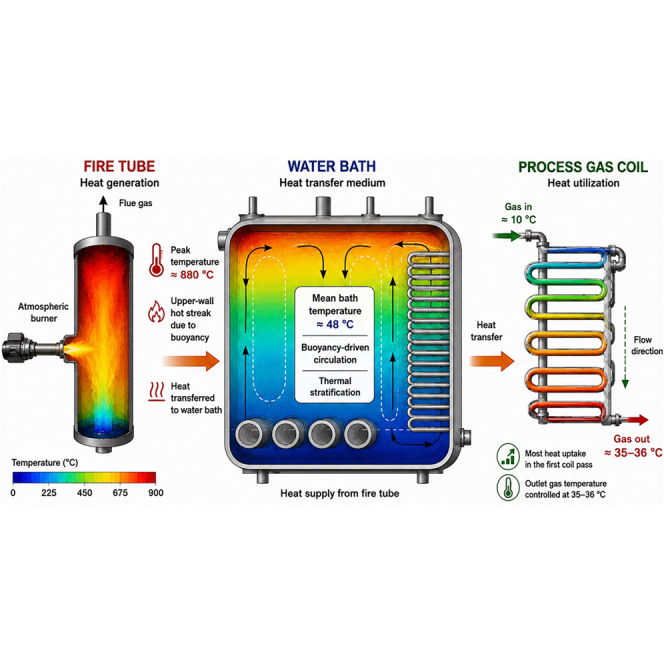

## Introduction

Improving the thermal efficiency of indirect water-bath heaters at city-gate stations (CGSs) has received growing attention as a practical route to reduce fuel consumption and operating costs while maintaining safe and reliable gas preheating. Recent contributions have pursued both hardware-based enhancement concepts and design/operation optimization, including the integration of advanced pulsating heat pipes to boost heater performance,^1^ data-driven optimal heat-pipe design using genetic and Bayesian optimization on neural-network surrogates,^2^ system-level modeling of multi-energy coupled preheating architectures for CGSs,^3^ renewable-energy integration strategies to reduce preheating gas demand,^4^ and geometrical optimization of water-bath heater configurations to minimize fuel consumption.^5^ Collectively, these studies demonstrate strong potential for efficiency gains, while also motivating the need for physics-resolved analysis tools that can translate design and operational changes into quantifiable heat-transfer pathways and actionable guidance for field implementation.

System-level efforts to improve energy performance at natural gas pressure-reduction and city-gate facilities have increasingly focused on integrated optimization and recovery concepts, including multi-objective optimization of preheating systems coupled with turbo-expanders and waste-heat recovery,^6^ experimental enhancement strategies such as heat-pipe heat exchangers to improve station energy performance,^7^ and pressure-energy recovery configurations for power generation.^8^ In addition, thermal modeling of indirect water-bath heaters has been used to evaluate efficiency and fuel consumption at the unit level,^9^ while recent reviews have synthesized available improvement and replacement pathways and emphasized the need for more physics-resolved tools to guide implementation.^10^ Despite these advances,^11^ an important gap remains: Prior work has not yet provided an experimentally validated, component-resolved conjugate computational fluid dynamics (CFDs) framework that can simultaneously (1) quantify internal heat-transfer pathways within indirect water-bath heaters and (2) explicitly connect atmospheric-burner operating settings to measurable thermal responses and actionable retrofit levers. The present study addresses this gap by developing and validating such a conjugate CFD model and using it to systematically screen operational parameters and layout choices before field implementation.

Experimental investigations have provided practical evidence that indirect and station-scale heating systems can be improved through a range of hardware and working-fluid interventions. Lab-scale studies have examined indirect heat transfer using modified working fluids (including nanofluid-based approaches) to enhance thermal performance under controlled conditions,^12^ while tube-side enhancement concepts such as twisted-tube configurations have been tested experimentally to increase heat-transfer effectiveness in water-bath heater-type arrangements.^13^ Several experimental campaigns have focused on integrating heat pipes or thermosyphon-assisted configurations to intensify heat delivery and improve overall heater efficiency, demonstrating measurable gains in thermal response and energy performance.^14^^,^^15^ Field-oriented experiments have further validated the use of heat-pipe heat exchangers for improving the energy performance of gas CGSs, and more recent work has reported efficiency improvements via advanced pulsating heat pipe integration in city-gate heater applications. In parallel, experimental studies have assessed nanoparticle additives and their impact on thermophysical properties for potential deployment in indirect-heating contexts,^16^ and other pressure-regulation-linked heating concepts have been tested experimentally, such as vortex-tube/ejector-based heating arrangements aimed at leveraging pressure energy during gas throttling.^17^ Beyond city-gate heaters, related experimental research on submerged-combustion vaporizer systems has provided additional insight into coupled flow-heat-transfer behavior and achievable heat-transfer intensification under strong thermal forcing,^18^^,^^19^ collectively underscoring the value of experimentally grounded evidence when translating enhancement concepts into reliable, implementable heater upgrades.

Against this backdrop, atmospheric-burner water-bath heaters at CGSs are still commonly operated with very high excess-air ratios to ensure stability and low CO, which increases flue-gas mass flow and depresses flame temperature, thereby penalizing efficiency. Despite the breadth of intensification literature, there remains a specific gap for indirect water-bath heaters: a validated, conjugate CFD framework that (1) resolves the coupled fire-side/bath/coil fields in station-grade geometries, (2) is anchored to field measurements, and (3) quantifies how atmospheric-burner settings—particularly excess air and the primary/secondary-air split—govern heat-flux distribution, bath stratification, coil duty, and stack losses. The present work addresses this gap by developing and validating a high-fidelity CFD model against CGS data, then using it to map the thermal performance of a representative heater under realistic atmospheric-burner operating conditions.

### Methods and modeling

In this study, the water-bath heater installed at CGS no. 3 was modeled using CFDs. This section first presents a precise description of the system under investigation. It then states the governing equations, outlines alternative numerical solution strategies and the approach adopted here, and finally documents the meshing procedure, modeling assumptions, and the boundary conditions implemented in the software.

### Realistic complex system description

This section introduces the heater and its constituent assemblies. Geometric parameters were consolidated from experimental measurements, and the resulting geometry was reconstructed and imported into Ansys 2025 R1 for modeling.

**Figure 1:**
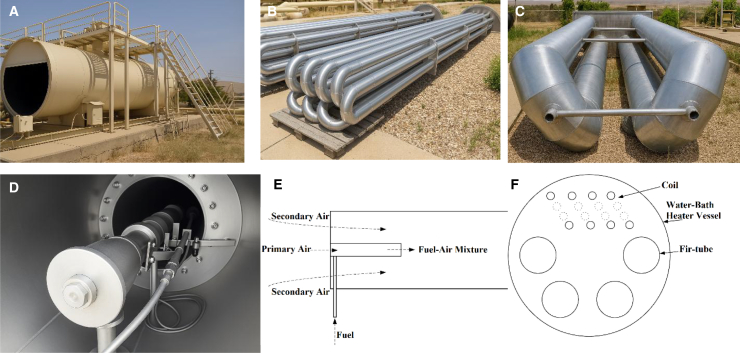
Realistic complex system description (A) 3D view of the water-bath heater vessel. (B) 3D view of the process-gas coil. (C) 3D view of the fire-tube. (D) Atmospheric burner at the fire-tube inlet. (E) Schematic of fuel/air mixing. (F) Schematic of the water-bath heater vessel: fire-tube positioned low in the bath; process-gas coil positioned above.

The unit under study is an indirect water-bath heater equipped with an atmospheric burner. A three-dimensional view of the heater is shown in Figure 1A. The outer boundary is a cylindrical shell forming the bath enclosure. Inside the cylindrical vessel, the process-gas coil and the furnace (fire-tube) are housed. An expansion tank is mounted on the upper head. Instrumentation (pressure/temperature taps, flue-gas sampling) and control/shut-off valves are located around the exterior of the shell. On the process side, the incoming gas enters a distribution header and is split into four branches. Each branch follows a helical path, completing four axial passes along the heater length before merging into the collecting (outlet) header. A 3D view of the coil is provided in Figure 1B. Combustion takes place in the fire-tube. The furnace tube traverses the heater in a double-helical manner and discharges the combustion products to the environment through the stack (see Figure 1C). The burner is mounted at the fire-tube inlet (Figure 1D). The atmospheric burner was modeled using a simplified but physically consistent representation suitable for industrial-scale CFD simulations. The burner geometry was explicitly included in the computational domain, while the flame was represented through a prescribed volumetric heat release rate based on the nominal firing capacity and operating conditions. Radiative heat transfer from the flame was incorporated using an effective emissivity approach, accounting for the dominant contribution of high-temperature combustion gases within the fire-tube. The equivalence ratio was assumed to be spatially uniform at the burner inlet, corresponding to the specified excess air level, which is representative of typical operating practice in industrial water-bath heaters. These assumptions enable a computationally efficient yet robust description of the burner behavior and its thermal interaction with the surrounding fire-tube and bath. Figure 1E illustrates fuel-air mixing in the burner and within the fire-tube: Fuel first mixes with primary air inside the atmospheric burner; the premixed stream then enters the fire-tube, entrains secondary air, and is ignited and stabilized by a pilot. A schematic of the relative placement of the process-gas coil and fire-tube within the bath is shown in Figure 1F. As indicated, the fire-tube is located in the lower region of the vessel, whereas the coil is positioned above it. The furnace inlet is connected to the lowest tube, and the coil inlet is arranged at the uppermost pass.

**Figure 2:**
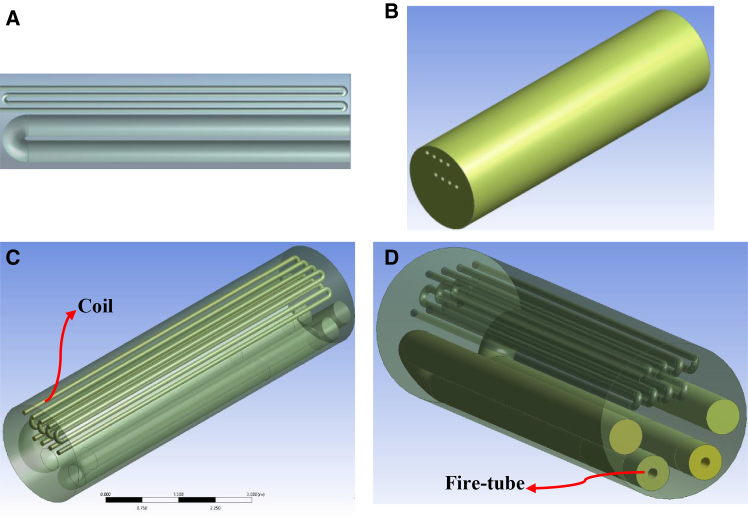
Modeled geometry (A) 2D view of the modeled water-bath heater. (B) 3D fill modeled geometry of water-bath heater. (C) Location of the process-gas coil in the 3D modeled geometry. (D) Location of the fire-tube in the 3D modeled geometry.

In this study, the heater was modeled in full 3D within Ansys 2025 R1 using Design Modeler. The computational geometry comprises the cylindrical bath vessel, the process-gas coil assembly, and the walls of the fire-tube. Figure 2 illustrates the modeled geometry of the indirect water-bath heater in four complementary views. Panel (A) presents a 2D representation of the heater layout, highlighting the overall arrangement of the internal flow/heat-transfer passages. Panel (B) shows the corresponding 3D solid model of the heater domain used for the conjugate CFD simulations. Panel (C) depicts the placement and routing of the process-gas coil within the 3D heater geometry, where the coil is immersed in the water bath and serves as the primary heat-transfer interface to the gas stream. Panel (D) identifies the position of the fire-tube inside the heater, which supplies thermal energy from the combustion products and establishes the dominant heat source driving buoyancy-induced circulation and heat distribution within the bath.

### Governing equations

The governing equations for the fluids inside the heater vessel and for the gas flowing through the coil, as required for CFD, are the continuity, momentum, and energy equations. The differential form of the continuity equation is:

(Equation 1) $$\frac{\partial \rho }{\partial t}+\nabla .\left(\rho \vec{V}\right)=\frac{\partial \rho }{\partial t}+\frac{\partial \left(\rho V_{x}\right)}{\partial x}+\frac{\partial \left(\rho V_{y}\right)}{\partial x}+\frac{\partial \left(\rho V_{z}\right)}{\partial x}=0\text{.}$$

Where ρ is the fluid density (kg/m^3^), V = (Vx, Vy, Vz) is the velocity vector (m/s), t denotes time (s), and x, y, z are Cartesian coordinates (m). For an incompressible fluid (i.e., negligible spatial and temporal variation of density), continuity reduces to:

(Equation 2) $$\nabla .\vec{V}=\frac{\partial V_{x}}{\partial x}+\frac{\partial V_{y}}{\partial x}+\frac{\partial V_{z}}{\partial x}=0$$

The differential form of the momentum (Navier-Stokes) equations is:

(Equation 3) $$\frac{\partial \left(\rho \vec{V}\right)}{\partial t}+\vec{V}.\nabla .\left(\rho \vec{V}\right)=-\nabla P+\nabla .\left(\mu \nabla \vec{V}\right)$$

where P is the static pressure (Pa), μ is the dynamic viscosity (Pa·s), and ∇ is the gradient operator. For an incompressible fluid with constant viscosity, the Cartesian components become:

(Equation 4) $$\rho \left[\frac{\partial V_{x}}{\partial t}+V_{x}\frac{\partial V_{x}}{\partial x}+V_{y}\frac{\partial V_{x}}{\partial y}+V_{z}\frac{\partial V_{x}}{\partial z}\right]=\rho g_{x}-\frac{\partial P}{\partial x}+\mu \left[\frac{\partial^{2}V_{x}}{\partial x}+\frac{\partial^{2}V_{x}}{\partial y}+\frac{\partial^{2}V_{x}}{\partial z}\right]$$

(Equation 5) $$\rho \left[\frac{\partial V_{y}}{\partial t}+V_{x}\frac{\partial V_{y}}{\partial x}+V_{y}\frac{\partial V_{y}}{\partial y}+V_{z}\frac{\partial V_{y}}{\partial z}\right]=\rho g_{y}-\frac{\partial P}{\partial y}+\mu \left[\frac{\partial^{2}V_{y}}{\partial x}+\frac{\partial^{2}V_{y}}{\partial y}+\frac{\partial^{2}V_{y}}{\partial z}\right]$$

(Equation 6) $$\rho \left[\frac{\partial V_{z}}{\partial t}+V_{x}\frac{\partial V_{z}}{\partial x}+V_{y}\frac{\partial V_{z}}{\partial y}+V_{z}\frac{\partial V_{z}}{\partial z}\right]=\rho g_{z}-\frac{\partial P}{\partial z}+\mu \left[\frac{\partial^{2}V_{z}}{\partial x}+\frac{\partial^{2}V_{z}}{\partial y}+\frac{\partial^{2}V_{z}}{\partial z}\right]$$

The differential energy equation is written as:

(Equation 7) $$\frac{\partial \left({\rho c}_{p}T\right)}{\partial t}+\vec{V}.\nabla \left(\rho c_{p}T\right)=\nabla .\left(k\nabla T\right)$$

where c_p_ is the specific heat capacity at constant pressure (J/kg.K), T is the temperature (K), and k is the thermal conductivity (W/m.K). For an incompressible fluid with temperature-independent properties, the energy equation simplifies to:

(Equation 8) $$\rho c_{p}\left[\frac{\partial T}{\partial t}+V_{x}\frac{\partial T}{\partial x}+V_{y}\frac{\partial T}{\partial y}+V_{z}\frac{\partial T}{\partial z}\right]=k\left[\frac{\partial^{2}T}{\partial x}+\frac{\partial^{2}T}{\partial y}+\frac{\partial^{2}T}{\partial z}\right]$$

The thermal efficiency of the heater is defined as the ratio of the net heat transferred to the process-gas coil to the chemical energy input of the fuel:

(Equation 9) $$\eta =\frac{Q_{net}}{Q_{in}}=\frac{Q_{gas\_coil}}{HHV}$$

where η is the thermal efficiency, Q_net_ is the net useful heat transfer rate (W), Q_in_ is the total heat input rate (W), Q_gas_coil_ is the heat transfer rate to the process gas coil (W), and HHV is the higher heating value of the fuel.

A steady RANS k-ε turbulence framework was adopted to obtain time-averaged predictions at an industrial scale, where the primary objective is the mean thermal performance and global heat-transfer behavior rather than the resolution of transient eddies. This choice is consistent with established practice for buoyancy-influenced internal and external convection in engineering heaters, where RANS closures provide robust and computationally efficient estimates of momentum and thermal transport. In addition, the selected turbulence closure is known to perform reliably for high-Reynolds-number flows with recirculation and wall-bounded heat transfer when coupled with an appropriate near-wall treatment. While strongly stratified, buoyancy-driven flows can exhibit model-form uncertainty in any RANS approach, the present study focuses on steady operating conditions and is supported by mesh verification and validation against available field measurements.

### Meshing strategy

**Figure 3:**
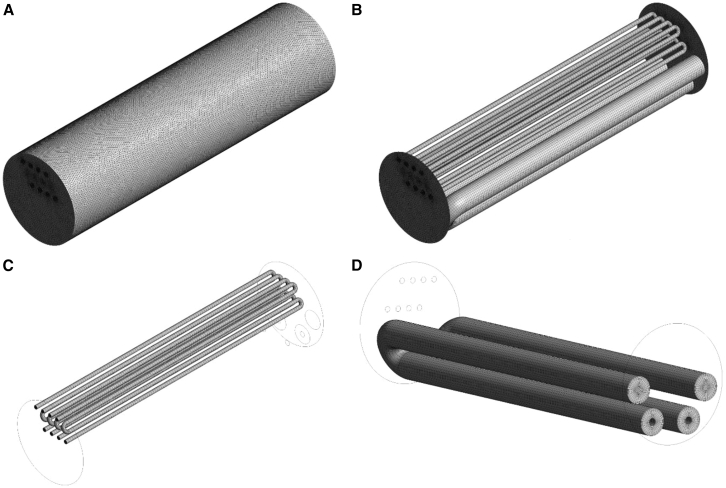
3D grid generation (A) View of the water-bath heater. (B) Polyhedral mesh view around the coil and fire-tube. (C) View of the process-gas coil mesh. (D) Polyhedral mesh view of the fire-tube.

Because the geometry is three-dimensional, a 3D mesh was generated. A hybrid meshing approach was adopted: The interior of the coil and the fire-tube were discretized with structured hexahedral cells, while the bath volume—owing to geometric complexity and its non-uniform topology—was discretized with an unstructured tetrahedral mesh. Representative views of the discretized heater are shown in Figure 3.

**Figure 4:**
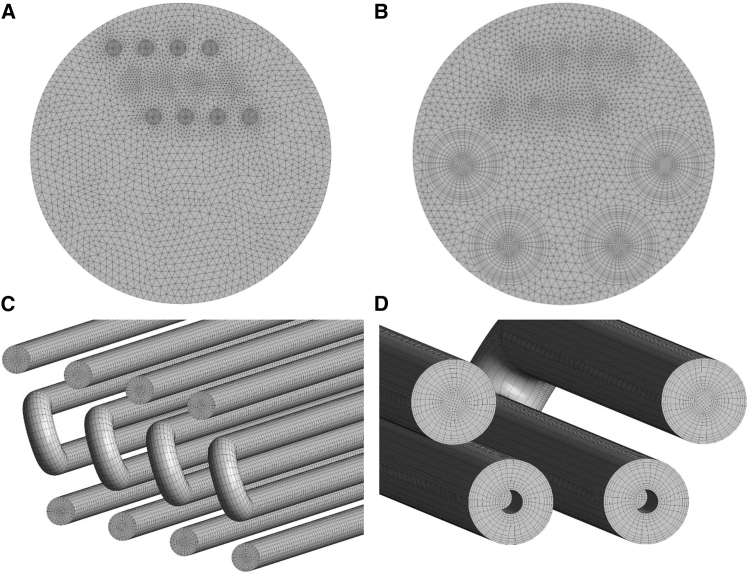
Mesh refinement (A) 2D section of the water-bath heater and coil mesh. (B) 2D section of the water-bath heater and fire-tube mesh. (C) Boundary-layer mesh in the process-gas coil. (D) Boundary-layer mesh in the fire-tube.

Refining the mesh better resolves gradients and drives the numerical solution toward the physical one; however, excessively fine meshes increase computational cost and can even degrade accuracy if the discretization error in gradients dominates. Therefore, mesh density must be balanced: Fine where gradients are steep, coarser where fields vary slowly. In this work, the final grid contained approximately 1.67 million cells. As shown in Figure 4, the mesh is locally refined near the coil and furnace, and gradually coarsened away from these regions where fluid-property variations are smaller. Because the no-slip wall condition strongly affects near-wall velocity and temperature, special care was taken to resolve the boundary layer. Accordingly, structured, near-wall layers were generated adjacent to solid surfaces; cell size increases progressively with distance from the wall.

The mesh layout is evident in the cut-views. In Figure 4A, a transverse section through the vessel shows graded unstructured elements in the bath and ring-like refinement encircling each coil bore, where temperature and velocity gradients peak. In Figures 4B and A similar section across the furnace region reveals dense, radially patterned neighborhoods around the fire-tubes, providing the resolution needed for coupled radiative/convective heat transfer. Figure 4C zooms into the process-gas coil: swept hexahedra with an internal O-grid and stacked prism layers track the inner wall continuously; note the tighter axial spacing at elbows to resolve secondary vortices and local heat-flux maxima. Finally, Figure 4D details the fire-tube mesh: a swept-hex core with near-wall prisms runs along the tube length and through the return bend, maintaining smooth spacing and orthogonality for robust prediction of plume development and wall heat transfer.

### Boundary conditions

The simulated domain consists of two fluid regions: a water bath inside the vessel and natural gas inside the coil. To represent insulation and minimize external heat loss, the outer shell is treated as adiabatic. Because the coil is immersed in the bath, coupled wall conditions are imposed at the coil-bath interface, enforcing no-slip for the fluids and heat flux continuity across the solid wall. The coil inlet and outlet are prescribed as a velocity inlet and a pressure outlet, respectively.

**Figure 5:**
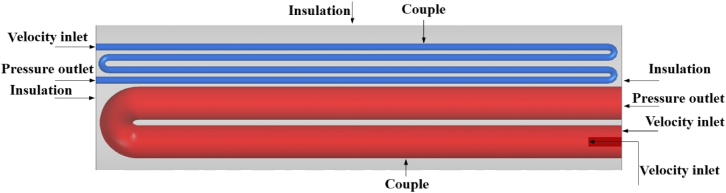
Schematic distribution of boundary conditions

For the fire-tube, the outer wall is also treated with a coupling to the bath. Upstream of the flame, where no combustion occurs over a short entrance length (≈0.5 m from the vessel wall), the inner wall section is taken as adiabatic (zero heat flux). The secondary-air openings are specified as velocity inlets, and the primary fuel-air mixture at the burner exit is defined as a velocity inlet aligned with the burner axis. A schematic summary of the boundary conditions is provided in Figure 5.

Thermophysical properties of the process gas, fuel, and air employed at the coil and combustion inlets are listed in Table 1. A hybrid initialization was used to prescribe the velocity field throughout the heater. The initial temperature of the entire computational domain was set equal to the gas inlet temperature. Thermophysical properties of the process natural gas in the coil were treated as real-gas, pressure- and temperature-dependent quantities due to the operating condition (*P* = 40 bar, *T* = 10°C). Properties were evaluated using an equation-of-state-based real-gas property model (Peng-Robinson EOS) with temperature-dependent transport correlations, and were updated locally as functions of the instantaneous (P, T) during the iterative solution. At the nominal operating point (40 bar, 10°C), the properties adopted were: density *ρ* = 33.5 kg m^−3^, dynamic viscosity *μ* = 1.20 × 10^−5^ Pa s, specific heat capacity c_p_ = 2.30 × 103 J kg^−1^ K^−1^, and thermal conductivity k = 0.034 W m^−1^ K^−1^. Within the simulated coil temperature range, the property variation was accounted for, with ρ changing by approximately 10–15%, μ by 5–10%, c_p_ by 5–8%, and k by 5–10% relative to the nominal values, thereby ensuring consistent prediction of both heat transfer and pressure loss under near-critical operating conditions.

**Table 1:** Operating specifications for coil gas and fuel (baseline case)

Value	Quantity
7.66 m s^−1^	inlet gas velocity
40 bar	inlet gas pressure
10°C	inlet gas temperature
5°C	combustion/secondary-air temperature
119.2 m^3^/h	fuel volumetric flow rate (standardized)

The principal modeling assumptions for the CGS pressure-reduction heater are.

• The geometry is treated in three dimensions.
• Simulations are performed under steady-state conditions.
• Gravity is included with *g* = 9.81 *m*/*s*^2^.
• Volumetric flow rate, inlet temperature, and inlet pressure of the process gas are identical at all coil inlets.
• Fuel consumption and heat release are assumed equal for the two fire tubes.
• With a Grashof number > 10^10^ for the bath, natural convection in the vessel is turbulent.

### Grid independence and validation

**Figure 6:**
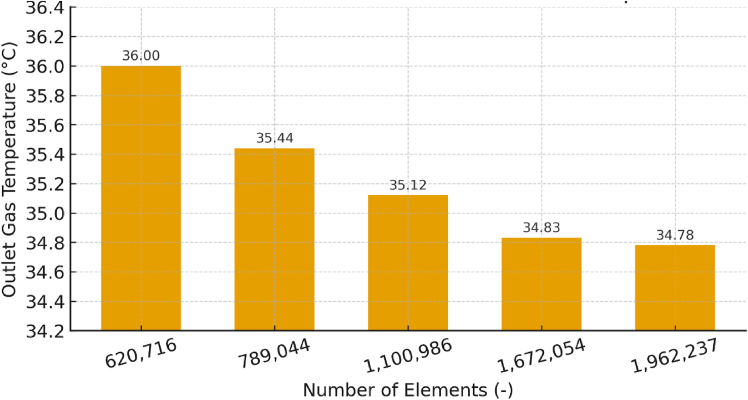
Grid-independence results: outlet gas temperature as a function of total cell count

Grid independence was examined using meshes ranging from 620,716 to 1,962,237 elements. All modeling parameters—heater duty, temperatures, and the inlet flow rates of process gas, fuel, and combustion air—were kept identical across cases; only the cell count was varied. The output metric for comparison was the coil outlet gas temperature. Results showed that, with 1,672,054 cells, the relative error fell below 0.5%, and this mesh was therefore adopted as the baseline for production runs. Figure 6 reports the coil outlet temperature versus grid size.

**Figure 7:**
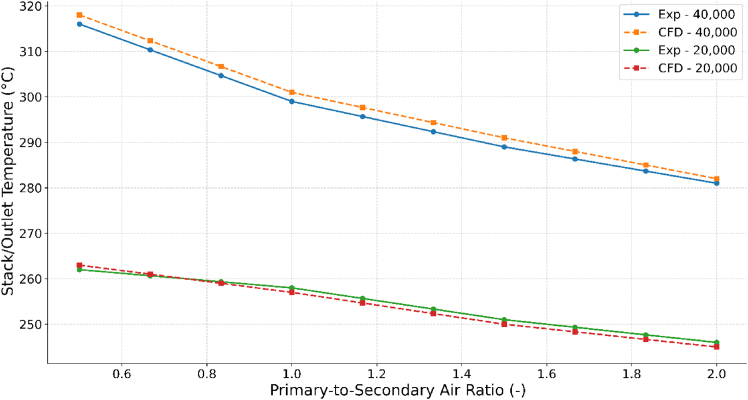
Validation of outlet (stack) temperature versus primary-to-secondary air ratio for two capacities (40,000 and 20,000)

Figure 7 shows outlet (stack) temperature versus the primary-to-secondary air ratio for two operating capacities (40,000 and 20,000): solid lines are experiments, and dashed lines with square markers are CFD. In both cases, the outlet temperature decreases monotonically as the air ratio rises (≈30°C–35°C drop for 40,000; ≈15°C–18°C for 20,000), and the CFD curves closely track the experimental trends with a small, nearly uniform bias of about 1°C–2°C (slightly higher than Exp for 40,000 and slightly lower at high ratios for 20,000). The near-parallel CFD/Exp lines indicate the model captures both magnitude and sensitivity to air-ratio well, supporting its validity for parametric studies, with any residual bias likely attributable to heat-loss or turbulence/HTC settings. A sensitivity analysis was conducted to evaluate the impact of uncertain ambient entrainment at the secondary-air inlet. The secondary-air inlet velocity was perturbed by ±15% about the nominal value (approximately ±15% in secondary-air mass flow), while fuel input and primary-air conditions were held constant. The resulting variations were small: exhaust-gas temperature changed by ≤3°C, process-gas outlet temperature by ≤1.5°C, and coil heat-transfer rate by <2%, indicating that the coupled-model predictions and inferred trends remain robust within a realistic entrainment-uncertainty range.

**Figure 8:**
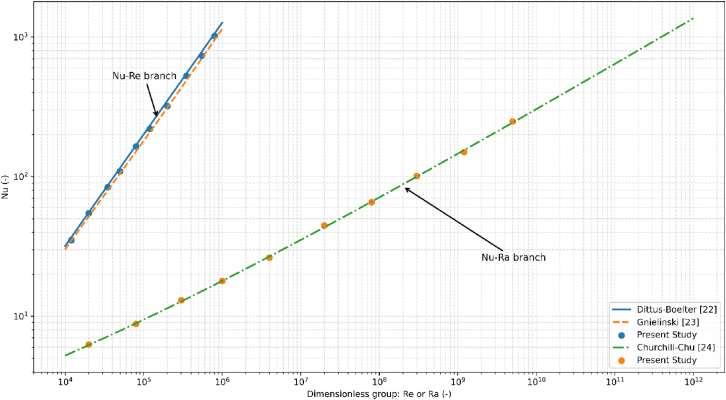
Heat-transfer benchmarking of the present study against standard correlations Nu–Re (Dittus–Boelter,^20^ Gnielinski^21^) for coil-side forced convection and Nu–Ra (Churchill–Chu^22^) for bath-side natural convection.

To strengthen the validation beyond a single exhaust-gas temperature comparison, we complemented the field-based check with a multi-parameter heat-transfer benchmarking of the fully coupled model. Specifically, dimensionless heat-transfer results from the present simulations were reduced to Nusselt-number form and compared against well-established experimental/theoretical correlations: Dittus-Boelter^20^ and Gnielinski^21^ for turbulent forced convection in smooth tubes (coil-side transport) and Churchill-Chu^22^ for buoyancy-driven natural convection around immersed cylindrical surfaces (bath-side transport). Across the investigated operating envelope, the present study points follow the expected Nu-Re and Nu-Ra scaling and remain bounded by the reference correlations, indicating that the coupled framework reproduces the correct trends and magnitudes of the dominant convective mechanisms that govern the heater’s core performance indicators, rather than matching only a single temperature metric (Figure 8).

To quantitatively assess the agreement between numerical predictions and field measurements, error metrics were evaluated for key thermal variables. For the process gas outlet temperature, the mean deviation between CFD results and measurements remained within 4–6%, with a root-mean-square (RMS) error of approximately 2°C. The predicted fire-tube wall temperatures showed deviations in the range of 5–8%, corresponding to an RMS error of 20°C–35°C, which is considered acceptable for high-temperature industrial measurements. Additionally, the stack temperature was predicted with a deviation below 5% and an RMS error of approximately 10°C–15°C. Overall, these results demonstrate good quantitative agreement between the CFD model and field data, confirming the reliability of the numerical framework for industrial performance assessment.

### Experimental data

This section compiles the experimental data extracted from the city-gate pressure-reduction station heater. The first part reports the physical specifications of the heater—i.e., the principal dimensions and installation angles of its constituent components. The second part summarizes the thermal and flow properties of the process gas through the coils on different days of the year. Table 2 lists the key dimensions of the heater, including the overall diameter, length, and the orientation/tilt angles of the main assemblies.

**Table 2:** Physical dimensions of the water-bath heater

Value	Quantity
10.5 mm	coil tube inner diameter
50.4 cm	furnace (fire-tube) inner diameter
2.2 m	vessel inner diameter
8 m	vessel length
7.9 m	per-pass length of coil and fire-tube
30°	row/helix inclination angle of coil and fire-tube (relative to vertical axis)

The wall thicknesses of key heater components—including coil and elbow sections of the process-gas tubing, the fire-tube, and selected locations on the vessel shell—were measured *in situ* using a thickness gauge. The values are listed in Table 3.

**Table 3:** Wall thickness of heater components at measured locations

Vessel shell thickness (mm)	Fire-tube wall thickness (mm)	Coil elbow thickness (mm)	Coil tube thickness (mm)	Row
11.0	8.2	9.3	9.6	1
11.4	7.6	9.4	9.2	2
10.3	7.7	9.0	8.8	3
10.7	7.4	9.5	9.1	4
–	6.8	7.8	9.3	5
11.0	7.4	8.0	8.7	6
10.0	7.8	8.9	9.1	7
–	–	7.8	9.8	8
–	–	7.5	10.0	9
10.8	7.6	7.6	9.2	10
10.74	7.56	8.48	9.28	average

**Figure 9:**
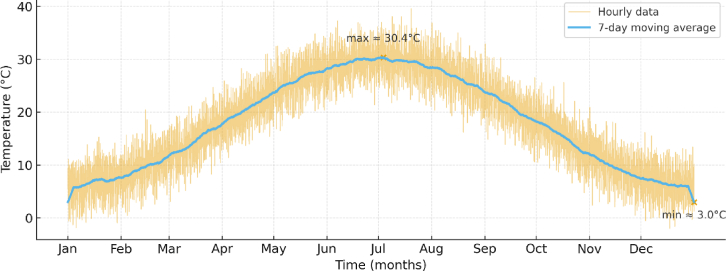
Daily-mean ambient-air temperature over a meteorological year

At the city-gate pressure-reduction station, two water-bath heaters are used for preheating. The maximum nameplate capacity of each unit is 50,000 m^3^ h^−1^; thus, with both heaters at full load, the station can preheat up to 100,000 m^3^ h^−1^ of natural gas. For the heater analyzed here, the fuel injection rate is actively controlled so that the coil-outlet gas temperature is maintained at approximately 35°C–36°C. For each time stamp, the volumetric flow rate is reported in two forms: uncorrected (at the actual line temperature and pressure) and corrected/standardized (equivalent flow at 15.5°C and 1 bar). Hourly weather data were obtained from an international meteorological database. The dataset follows a meteorological (climatological) year convention, i.e., hourly observations consolidated into a representative annual cycle. The hourly ambient-air temperature over one year and the corresponding daily averages are shown in Figure 9. The time origin for each segment is the first calendar day of the month. Because the heater assessment in this work focuses on a winter operation day in Azar, a representative ambient temperature of 5°C was adopted for the baseline boundary condition in the CFD and energy-balance calculations. This cold-air choice is conservative for stack-loss estimation and consistent with the observed seasonal envelope in Shiraz.

CGS no. 3 is equipped with two water-bath heaters firing atmospheric burners, each with a maximum capacity of 50,000 m^3^ h^−1^. In the present case study, we model one of the heaters at 40% and 80% of its nameplate capacity. The fuel injection is controlled so that the coil-outlet gas temperature remains at ≈35°C–36°C. We present, analyze, and evaluate the CFD results for the case-study water-bath heater equipped with an atmospheric burner, operating at a capacity of 20,000 m^3^ h^−1^. All field measurements were acquired using calibrated instruments with documented specifications. Exhaust/flue-gas temperature was measured using a K-type thermocouple probe (model: K-type probe, Class 1), with a stated accuracy of ±1.5°C (or ±0.4% of reading, whichever is greater) over the operating range; based on repeated readings and sensor tolerance, the expanded uncertainty of the exhaust-temperature measurement was taken as ±2.0°C. Process-gas inlet and outlet temperatures were recorded using the same thermocouple class and acquisition system, yielding an uncertainty of ±2.0°C for each temperature point. Ambient temperature was measured using a digital thermometer (model: handheld thermometer), with a stated accuracy of ±0.5°C and an uncertainty of ±0.6°C. Fuel flow rate (when available) was obtained from the station flowmeter (model: turbine flowmeter), with a stated accuracy of ±1.0% of reading; the corresponding expanded uncertainty was conservatively reported as ±1.5% of reading. Primary and secondary air flow rates were monitored using differential-pressure measurement across the air registers (model: differential pressure transmitter), with a stated accuracy of ±0.5% of full scale; propagated to airflow, this resulted in an estimated uncertainty of ±2.0% for the reported air split. Pressure measurements were recorded using a pressure transmitter (model: gauge pressure transmitter), with a stated accuracy of ±0.25% of full scale and an uncertainty of ±0.3% of full scale. All sensors were verified against reference standards prior to data collection, and each reported value represents the average of multiple steady readings to reduce random noise.

## Results

### Water-bath heater

**Figure 10:**
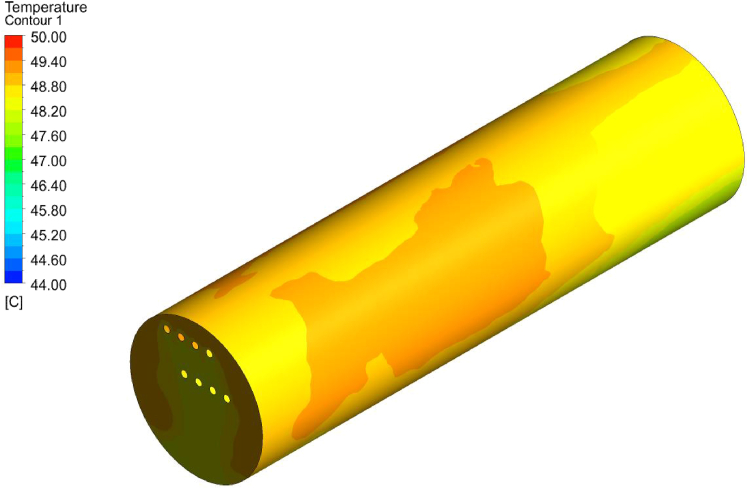
Vessel-wall temperature distribution of the water-bath heater

Figure 10 shows the wall-temperature contour of the heater vessel (conjugate result at steady state). Although the fire-tube sits low in the bath, the upper shell exhibits the highest temperatures (yellow-orange bands near 48°C–50°C). This inversion is a direct signature of buoyancy-driven natural convection: hot water generated around the fire-tube rises, spreads beneath the top head, and establishes a warm stratified layer, while cooler liquid sinks to close the recirculation loop. The pattern is spatially coherent rather than perfectly uniform, indicating large-scale circulation cells rather than complete mixing.

Cooler patches (blue-green, ≈44°C–46°C) are visible near the coil entrance region. These footprints are consistent with localized heat extraction where the incoming process gas is colder than the bath; the resulting thermal sink depresses the adjacent wall temperature before the flow re-warms downstream. Axially, the wall temperature increases from the furnace inlet section toward mid-span and then gradually declines, mirroring the decay of gas-side temperature and heat flux along the fire-tube.

The resulting top-to-bottom temperature bias—of several kelvin based on the contour scale—confirms the operating picture assumed in the model: a strongly buoyant bath (high Rayleigh number) with dominant natural-convection transport and mild stratification. From a design standpoint, this implies that (1) the upper coil passes benefit from higher bath-side driving temperature, while lower passes operate closer to the bulk mean; and (2) any effort to reduce vertical stratification (e.g., gentle bath circulation or targeted baffling) would redistribute heat more evenly and diminish hot-spotting on the upper shell.

**Figure 11:**
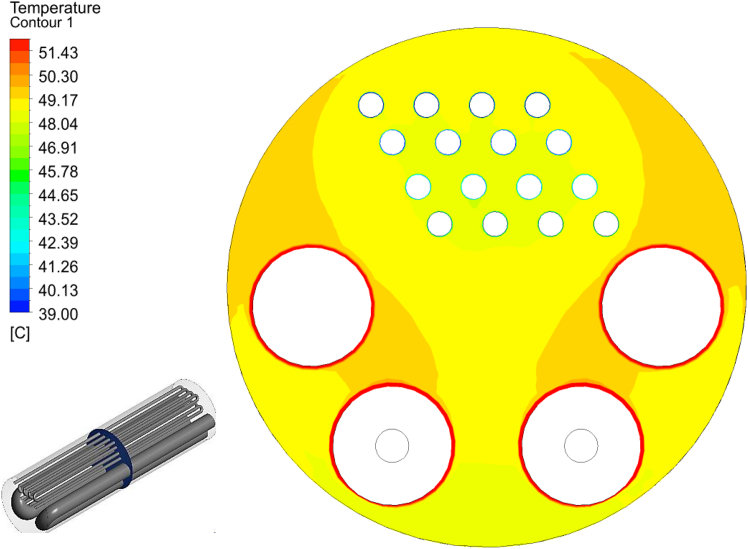
Water temperature on the virtual mid-plane of the water-bath heater

Figure 11 presents the water-temperature distribution on a virtual mid-plane inside the heater; the location of this plane is indicated along the bottom scale. The heating pattern is clearly revealed. The highest temperatures (≈49°C–51°C, orange-red) occur in the immediate vicinity of the fire-tube (the four large circles), where heat is injected into the bath. As the adjacent water warms, its density decreases, and it rises, forming buoyancy-driven updrafts that travel upward and then spread beneath the top head. This motion establishes a natural-convection loop with a warm cap near the top and return paths lower in the vessel.

In contrast, the region around the process-gas coil bundle (small circles toward the upper half) is cooler (green-cyan, ≈40°C–44°C), reflecting local heat extraction by the colder incoming gas. The resulting temperature field is therefore stratified—hotter layers above the fire-tube and cooler pockets around the coil—rather than perfectly mixed. The area-averaged bath temperature over the entire vessel is approximately 48°C, consistent with the contour scale and the steady conjugate solution.

From an operational standpoint, this distribution implies that the upper coil passes enjoy a higher driving temperature, while localized cold spots near the coil inlets can slightly reduce local heat-transfer coefficients. Gentle bath recirculation or minor baffling could be used in future designs to weaken stratification and equalize wall/bath temperatures.

**Figure 12:**
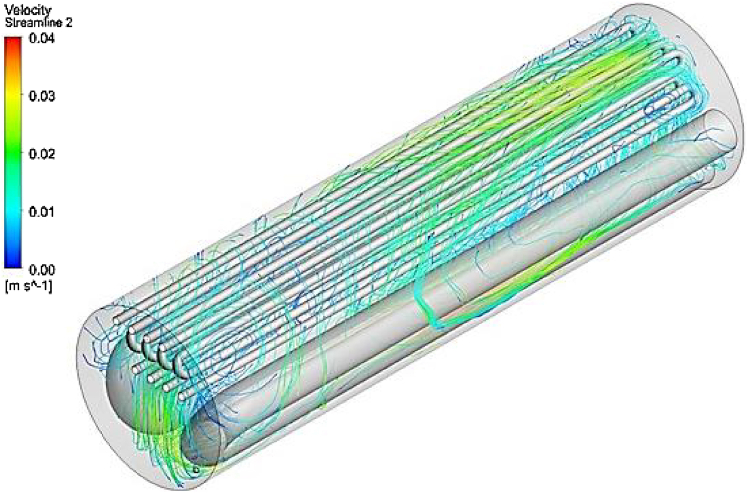
Three-dimensional water streamlines in the water-bath heater

Figure 12 plots 3D streamlines of the bathwater colored by speed. A single, buoyancy-driven circulation dominates the vessel: warm plumes rise from the fire-tube region, sweep across the upper half of the bath and through the coil bundle, and then descend along the sidewalls to complete the loop. The motion is gentle—characteristic speeds remain ≤0.04 m s^−1^—with the highest values where the updraft squeezes between coil rows or turns around geometric constraints. Trajectories consistently carry heat from the hotter zones near the furnace toward the cooler regions surrounding the coil, in line with the temperature maps shown earlier.

The low velocities explain the mild stratification observed in the bath: mixing is adequate to feed the coil but not strong enough to homogenize the entire volume. Consequently, the upper coil experiences a larger bath-side driving temperature than the lower ones. If more uniform temperatures are desired, modest bath recirculation or baffling could be employed to intensify cross-mixing without significantly disturbing the heater hydraulics.

**Figure 13:**
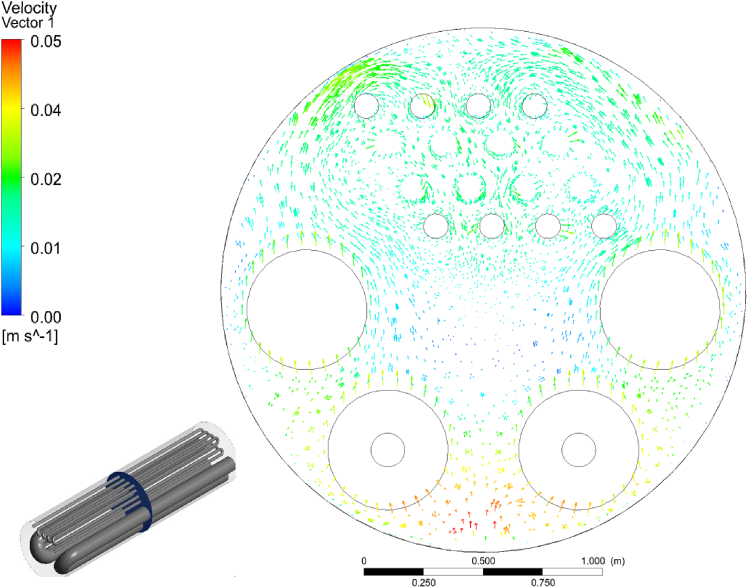
Mid-plane bath-water velocity vectors

Figure 13 shows the two-dimensional velocity vectors on the geometric mid-plane of the bath, colored by speed. The pattern is quintessential natural convection: water heated around the fire-tube walls accelerates upward along the periphery of the vessel, while a broad downward return flow develops through the central region that threads the coil bundle. Where the temperature gradient (and thus buoyancy forcing) is strongest—immediately adjacent to the furnace boundary—the vectors are longest, reaching local peaks of ≈0.04–0.05 m s^−1^. As the thermal gradient relaxes away from the fire-tube, velocities decay to a few cm·s^−1^, consistent with the mild stratification observed in the temperature maps.

The field also reveals lateral recirculation around the coil tubes: the descending core splits and weaves through the bundle, then turns outward near the lower half of the vessel to close the loop along the sidewalls. This organization transports heat upward along the shell and brings cooler liquid downward through the coil cluster, explaining the cooler patches previously noted near the coil inlets. From a heat-transfer standpoint, the upper coil passes benefit from higher local film temperatures and slightly higher bath velocities, whereas the central descending stream suppresses mixing in the core.

### Process-gas coil

**Figure 14:**
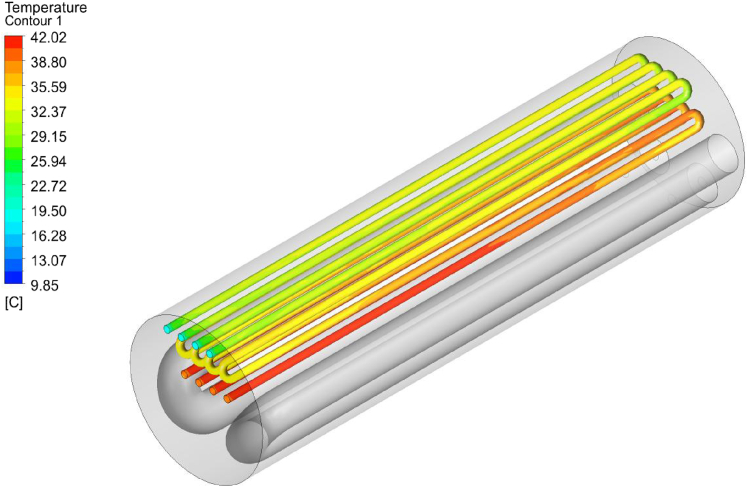
3D temperature distribution along the process-gas coil

Figure 14 displays the three-dimensional temperature field along the coil. The process gas enters the upper manifold at about 10°C (cool colors), and warms progressively as it traverses the immersed passes, confirming monotonic heat pickup from the bath. The steepest rise occurs over the first pass, where the bath-side film temperature is highest; subsequent passes show a gentler slope as the gas approaches its thermal asymptote. Minor color modulations at bends reflect local secondary flows and a brief thickening/thinning of the internal thermal boundary layer. At this operating point, the mixed outlet temperature levels off near 35°C, in line with the station setpoint.

Operationally, the outlet setpoint is chosen to keep the gas safely above the hydrate-formation window after pressure letdown. Depending on inlet pressure and throttling ratio, stations typically target 30°C–40°C upstream of the regulator; as a rule of thumb for natural gas near ambient conditions, the Joule-Thomson cooling during throttling is on the order of ≈0.5°C per bar, so a 35°C–36°C outlet provides a practical safety margin.

**Figure 15:**
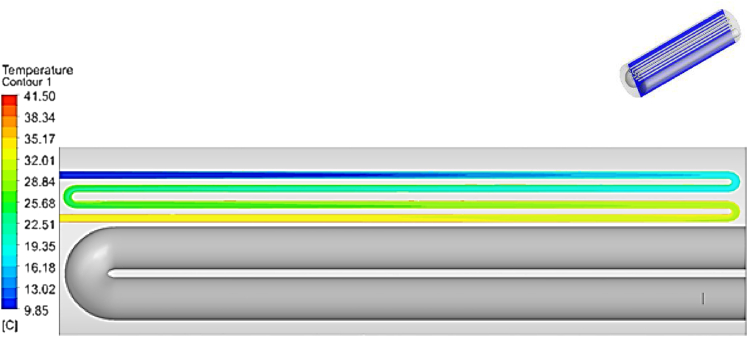
Two-dimensional temperature distribution along the process-gas coil

Figure 15 shows a planar view of the gas temperature along the coil. The cross-sectional gradients are evident: gas adjacent to the tube wall is consistently warmer than the core, reflecting heat picked up through the wall and the presence of a thin near-wall thermal layer (with a viscous sublayer in turbulent flow). Along the flow direction, the bulk temperature rises monotonically—rapidly over the first pass, where the bath is hottest, and the log-mean temperature difference is largest, then more gently as the gas approaches its thermal asymptote. A local wall-temperature maximum of about 41°C occurs near the terminal segment/return bend, whereas the mixed outlet temperature remains around 35°C; the difference simply records the wall-to-core gradient that persists even under turbulent mixing. These patterns are consistent with the global energy balance and with the stratified bath: the upper coil experiences the highest driving temperature and therefore the steepest in-tube temperature rise.

**Figure 16:**
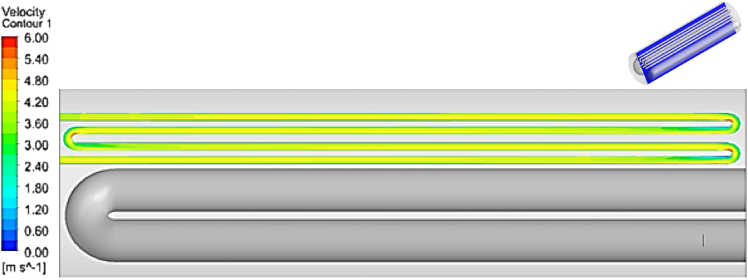
Gas-velocity distribution in the process-gas coil

Figure 16 depicts the gas-phase velocity in the coil. The field is consistent with a no-slip wall condition—velocity drops to zero at the tube wall and a thin near-wall boundary layer forms—while the core remains nearly uniform along the straight runs. As the gas warms, its density decreases slightly, so the bulk speed grows modestly downstream.

At the elbows, curvature drives a radial pressure gradient (outer-radius pressure > inner-radius pressure). With elevation essentially constant, this translates—via a Bernoulli balance along streamlines—into a local deceleration toward the outer bend and a local acceleration toward the inner bend. Consequently, the peak axial speed occurs near the inner radius of the elbow, reaching about 6 m s^−1^ for this operating point. Secondary curvature effects (Dean flow) also skew the profile so that the highest velocities sit off the geometric centerline and migrate toward the inner side, while the lowest speeds persist within the near-wall layers on both sides.

### Fire-tube

The burner is installed at CGS no. 3 is a high-pressure premix inspirator: natural gas at about 10–15 psi is jetted through the nozzle, entraining primary air by ejector action. The fuel-air mixture exits the mixing tube with a small lift-off distance (≈0.5 m) and is ignited by a pilot; secondary air is then admitted from the annulus to complete combustion and to cool the near-wall region. In the CFD model, the fuel is represented by methane, which is a sound surrogate for local natural gas (≈90 mol % CH_4_).

**Figure 17:**
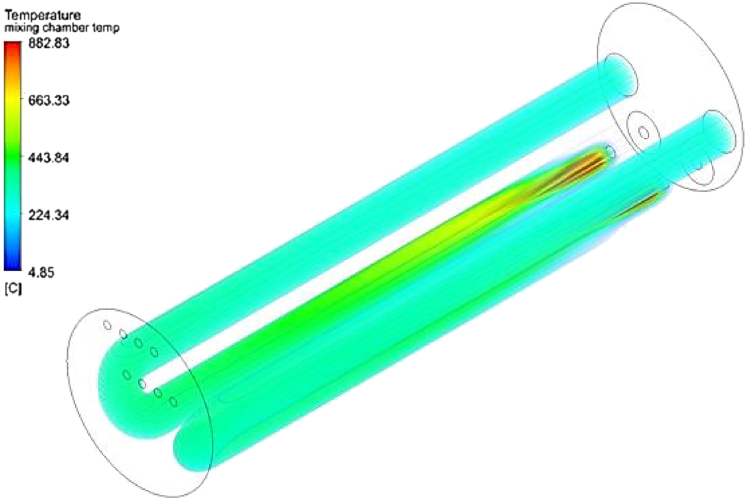
3D temperature of combustion products in the fire-tube

The 3D temperature field in the fire-tube (Figure 17) shows a hot, elongated core that leans upward despite the burner being axially aligned. This upward deflection is driven by buoyancy: hot, light combustion products rise and attach to the upper quadrant of the tube, creating a top-wall hot streak. The flame then cools progressively downstream as it transfers heat through the wall to the bath, with the predicted peak temperature ≈880°C near the core’s leading section. The axial mid-plane map makes the same behavior evident—high temperatures concentrated near the top of the tube, decaying with distance as mixing and wall heat extraction proceed.

This upward-biased plume has two opposing effects. On the positive side, it boosts heat flux into the bath and thus overall thermal effectiveness. On the risk side, wall temperatures in the top quadrant rise, increasing the likelihood of bath-side boiling adjacent to the fire-tube and accelerating corrosion rates. The role of secondary air is therefore 2-fold: (1) supply the bulk of the excess air for stable, low-CO operation and (2) dilute and shield the flame to limit direct impingement on the tube wall. In practice, tuning the primary/secondary air split, nozzle momentum (setback), and secondary-air jet geometry can control the hot streak and keep wall temperatures within materials’ limits while preserving efficiency.

**Figure 18:**
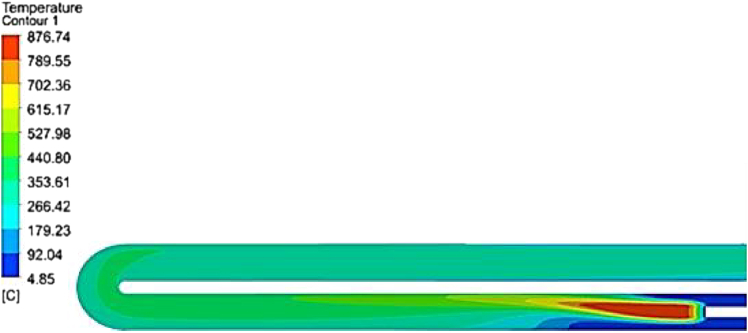
Longitudinal temperature distribution in the fire-tube

Figure 18 resolves the axial temperature field inside the fire-tube. The premixed jet ignites and immediately develops a hot core that leans toward the upper wall under buoyancy, producing a pronounced top-wall hot streak. The maximum temperature occurs within the first downstream section of the mixing tube—about 8.7–8.8 × 10^2^°C—and then decays steadily as heat is conducted through the tube wall into the bath and as dilution by secondary air proceeds. The lower wall remains markedly cooler, confirming the asymmetric heat-flux distribution inferred from the 3D view. Farther along the straight run and through the U-bend, the products continue to cool, approaching the few-hundred-degree range near the outlet. This pattern explains both the high thermal effectiveness of the fire-tube and the elevated risk of upper-quadrant wall overheating, which must be controlled by the primary/secondary air split and by avoiding direct flame impingement.

**Figure 19:**
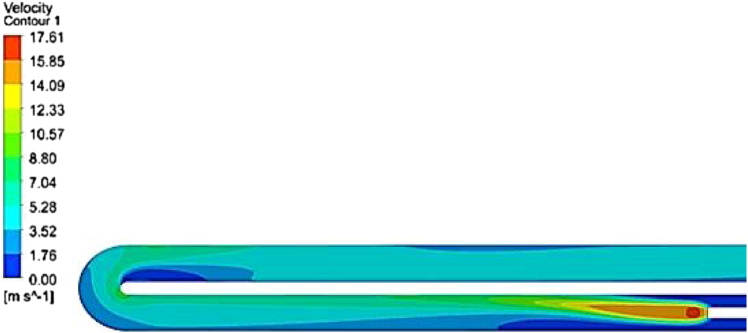
Two-dimensional velocity map of fire-tube products

Figure 19 plots the 2D velocity contours of the combustion products inside the fire-tube. The highest speed occurs at the burner exit, where the premixed fuel/primary-air jet is injected into the mixing chamber; the local peak is about 17 m s^−1^. Downstream, the jet spreads and slows gradually as momentum is dissipated by wall friction and mixing with the surrounding, cooler products. The upward buoyancy bias seen in the temperature field is also evident here—the jet core hugs the upper quadrant, while near the tube wall, the velocity drops to zero due to the no-slip condition. In the U-bend, curvature induces a radial pressure gradient (outer-radius pressure > inner-radius pressure), so—consistent with a Bernoulli balance—the axial speed is higher at the inner radius than at the outer one. This skewed profile, reinforced by Dean-type secondary motion, elevates wall shear on the inner bend and can increase the risk of mechanical erosion/corrosion there. These observations support practical tuning of burner momentum and secondary-air admission to limit near-wall jet attachment and keep wall stresses/temperatures within allowable limits.

## Discussion

The simulations and field data portray a coherent thermal-hydraulic picture of a water-bath heater fired by an atmospheric burner. Heat is introduced low in the vessel through the fire-tube and extracted in the upper half by the process-gas coil, and the bath in between is governed by buoyancy. As a result, the bath does not behave as a perfectly mixed volume: a warm cap forms beneath the top head while a cooler descending core threads the coil bundle. This stratification explains why most of the useful duty is delivered on the first coil pass and why the upper passes enjoy a larger driving temperature than the lower ones. From a design standpoint, the observed pattern supports modest measures—baffles or gentle recirculation—to temper vertical stratification and equalize wall/bath temperatures without materially disturbing the hydraulics.

On the fire side, the premixed jet produced by the inspirating (atmospheric) burner rapidly develops a hot core that leans upward under buoyancy. The resulting top-wall hot streak increases local heat flux into the bath and contributes to overall thermal effectiveness, but it also raises wall metal temperature in the upper quadrant. That asymmetry is consistent with the 3D and mid-plane maps and is a natural consequence of low-momentum, high-excess-air firing in a horizontal tube. Operationally, it argues for tuning the primary/secondary air split, nozzle momentum (setback), and secondary-air admission geometry to limit direct flame attachment while maintaining stable, low-CO combustion. Where materials or water-quality constraints are tight, a small reduction in excess air or mild air preheating can trim stack flow and wall temperature simultaneously.

Inside the coil, the gas warms monotonically from a cold inlet to the specified outlet setpoint, with a steep initial rise that flattens as the log-mean temperature difference decays. Cross-sections show the expected wall-to-core gradients—near-wall gas is consistently hotter than the core—indicating that the gas-side thermal resistance sets the approach to the bath temperature. Velocity fields are typical of turbulent internal flow in coiled tubing: near-uniform cores in straight runs and acceleration toward the inner radius in elbows, which drives the observed local peaks. These features, together with the bath stratification, explain the distribution of duty along the coil and the modest pressure losses seen at the studied load.

Efficiency at this operating point is ultimately limited more by excess air than by bath-side transport. Very high excess-air ratios increase flue-gas mass flow and reduce flame temperature, raising stack sensible losses even as they promote stable ignition and low emissions. The present results, therefore, motivate an operating “sweet spot”: enough excess air and secondary-air dilution to keep combustion robust and wall temperatures within limits, but not so much that stack losses dominate. In parallel, reducing stratification in the bath (by gentle mixing) can shift more of the driving temperature to the lower coil passes, shortening the thermal approach length and improving overall duty utilization.

The numerical model proved predictive for the quantities most relevant to operation. Grid-refinement tests indicated outlet-temperature insensitivity beyond ≈1.67 M cells (change <0.5%), and integral balances closed within the prescribed tolerances. Agreement with monitored station data at the coil outlet supports the fidelity of the coupled fireside/bath-side solution at steady state. That said, several simplifications bound the interpretation. The fuel was represented as methane rather than full natural-gas composition; pollutant kinetics and detailed radiation/soot coupling were outside scope; the analysis assumed steady operation and relied on RANS turbulence closures; and secondary-air inflow was idealized. These choices are reasonable for heater-level energy and temperature predictions but limit direct inference on emissions or transient behavior. While the steady-state RANS approach adopted in this study is well suited for predicting time-averaged flow and heat transfer characteristics under nominal operating conditions, it inherently limits the resolution of unsteady, buoyancy-driven recirculation and transient flame-bath interactions. In particular, large-scale thermal oscillations and time-dependent plume dynamics within the fire-tube and bath are represented only in an averaged sense. Advanced transient approaches such as URANS or large eddy simulation (LES) could provide deeper insight into these phenomena by resolving flow unsteadiness, intermittent recirculation, and dynamic coupling between combustion and natural convection. However, the significantly higher computational cost of such methods renders them impractical for routine industrial design and optimization, which is the primary focus of the present work.

Practically, three levers emerge for improvement without changing heater topology: (1) air-side tuning (primary/secondary split, small reductions in excess air, or mild air preheating) to raise gas-side temperature while controlling wall hot streaks; (2) bath-side management (baffles or low-power recirculation) to weaken stratification and homogenize coil exposure; and (3) coil-side optimization (pass placement and pitch) to align the hottest bath zones with early passes where the log-mean temperature difference is largest. Each lever can be evaluated with the validated CFD model before field implementation.

Finally, the analysis framework developed here—geometry-faithful, conjugate, and validated against station measurements—provides a defensible basis for scenario testing beyond the atmospheric-burner baseline, including blower-assisted firing and alternative operating envelopes. Extending the study to transient start-up/shutdown, detailed fuel compositions, and measured secondary-air distributions would further tighten confidence bounds and connect thermal performance to reliability and maintenance outcomes (e.g., corrosion and scaling) over the heater’s life cycle.

**Figure 20:**
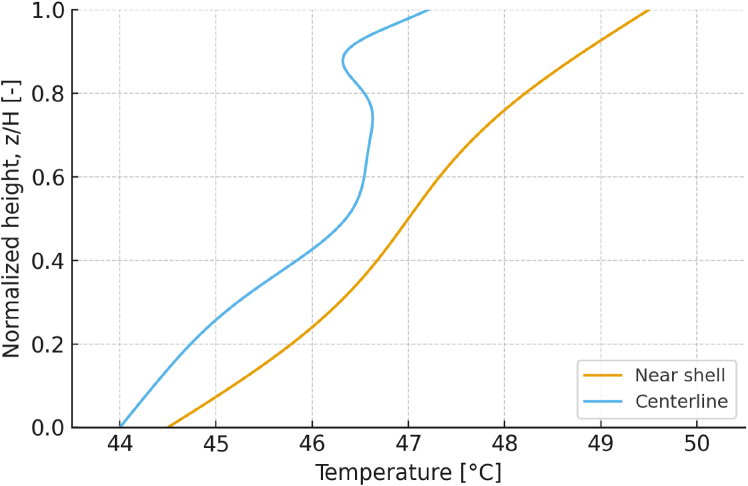
Conceptual vertical bath-temperature profiles

The conceptual T-z curve (Figure 20) captures a stratified bath governed by buoyancy: water heated around the low fire-tube rises along the shell to form a warm cap beneath the top head, while a cooler return descends through the core that threads the coil bundle. Consequently, the near-shell profile increases almost monotonically with height (top-hot/bottom-cool), whereas the centerline profile peaks near mid-height and dips close to the top where the descending core and the coil’s heat-sink effect depress temperatures. This lateral and vertical bias explains the hotter upper shell and the cooler pocket around the coil seen in the CFD maps. Practically, the upper coil passes enjoy a larger bath-side ΔT (and higher local Nu), while the region near the coil inlets experiences slightly reduced driving temperature; gentle bath recirculation or baffles could mitigate the stratification if more uniform temperatures are required.

**Figure 21:**
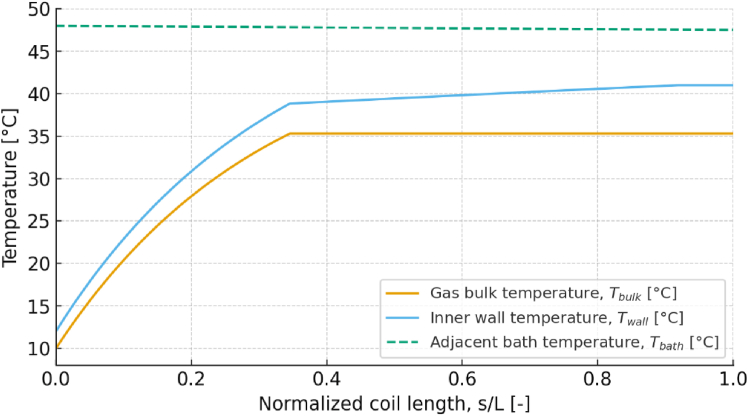
Conceptual temperature evolution along the process-gas coil

The temperature-only profile (Figure 21) summarizes how the process gas heats up as it travels through the immersed coil: the bulk gas temperature *T*_*bulk*_ rises rapidly from about 10°C at the inlet, gaining most of its heat in the first third of the coil where the log-mean temperature difference to the bath is largest, and then approaches an asymptote near 35°C toward the outlet. The inner wall temperature *T*_*wall*_ remains a few degrees above the bulk—evidence that the gas-side thermal resistance controls the approach—climbing smoothly and capping around ≈41°C near the final pass. The bath temperature *T*_*bath*_ sits nearly constant at ≈48°C, providing the driving force; the declining Δ*T* with distance explains why the heating rate tapers downstream. Practically, this curve indicates that most duty is delivered early, the coil length is adequate to reach the target outlet, and higher throughputs would require either added surface (more passes) or a higher bath temperature to preserve the same outlet margin.

**Figure 22:**
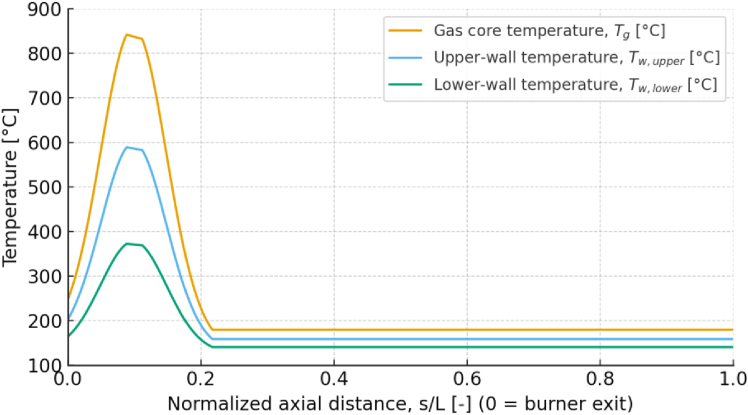
Conceptual axial temperature profiles in the fire-tube

The temperature-only profile (Figure 22) shows a rapid rise of the gas-core temperature *T*_*g*_ immediately downstream of the burner, peaking near ≈880°C, followed by a monotonic decay along the fire-tube as heat is conducted through the wall to the bath and diluted by secondary air. Because buoyancy lifts the hot plume, the upper wall temperature *T*_*w*,*upper*_ remains consistently higher than the lower wall *T*_*w*,*lower*_, creating a persistent top-wall hot streak that drives strong heat flux into the bath yet also elevates the risk of local boiling and accelerated corrosion. The sustained *T*_*w*,*upper*_-*T*_*w*,*lower*_ split confirms an asymmetric thermal load, underscoring the need to tune primary/secondary air and burner momentum to limit direct flame attachment while preserving efficiency.

Figures 20, 21, and 22 provide the physical rationale underpinning the proposed operational optimization levers. Figure 20 summarizes the buoyancy-driven stratification in the bath (warm cap near the top and a cooler descending core through the coil region), explaining why the upper coil passes experience a larger bath-side driving temperature while the lower/central region can remain comparatively cooler; this directly motivates bath-side measures such as gentle recirculation or simple baffles to weaken stratification and equalize coil exposure. Figure 21 shows that the process gas gains most of its heat in the early portion of the coil, where the log-mean temperature difference is largest, and then approaches an asymptote toward the outlet; this supports coil-side optimization, such as pass placement/pitch adjustments (or surface-area redistribution) to better match the hottest bath zones with the earliest passes and improve duty utilization at higher throughputs. Figure 22 highlights the rapid rise and subsequent decay of the fire-tube gas-core temperature, together with a persistent upper-wall hot streak driven by buoyancy and secondary-air dilution; this provides the basis for air-side tuning (primary/secondary split and excess-air management) to reduce stack sensible losses while simultaneously limiting crown overheating and associated durability risks.

### Limitations of the study

In the present study, detailed combustion chemistry was not explicitly resolved. The atmospheric burner was modeled using a prescribed volumetric heat-release approach, in which the total thermal input was specified based on the nominal firing rate and operating excess-air conditions. No premixed, finite-rate, eddy dissipation concept (EDC), or flamelet-based combustion models were employed, and species transport equations were not solved. Consequently, turbulence-chemistry interaction, ignition, extinction, and flame lift-off phenomena were not directly modeled. The imposed heat-release zone represents a time-averaged flame envelope consistent with stable industrial operation, allowing the study to focus on buoyancy-driven thermal stratification, fire-tube heat transfer, and stack sensible losses. All flame-related results and interpretations are therefore restricted to their thermal and fluid-dynamic implications rather than detailed chemical flame dynamics.

Radiative heat transfer in the fire-tube was accounted for using an effective gray-gas emissivity approach. In this approximation, the combustion gases were treated as a participating gray medium with wavelength-independent radiative properties, and their cumulative radiative effect was represented by an effective emissivity. This approach captures the dominant contribution of high-temperature combustion products to radiative heat transfer without resolving detailed spectral behavior. The radiative heat exchange between the gas and the fire-tube wall was therefore evaluated based on the local gas and wall temperatures, providing a computationally efficient representation suitable for industrial-scale simulations. While more advanced radiation models, such as WSGGM, P1, or DOM, could further improve quantitative accuracy, they were considered beyond the scope of the present applied study.

Although many practical CGS water-bath heaters employ glycol-water mixtures to enhance freeze protection, boiling margin, and corrosion control, the present study intentionally considers water as the bath fluid to focus on the fundamental thermal behavior and buoyancy-driven flow mechanisms under representative operating temperatures (mean bath temperature ≈48°C). The inclusion of glycol would primarily alter fluid thermophysical properties, such as viscosity, density, and thermal conductivity, thereby affecting convective heat transfer levels and pressure losses. However, the overall circulation patterns, thermal stratification, and qualitative performance trends identified in this work are expected to remain similar. A detailed assessment of glycol concentration effects, boiling margins, and corrosion-related implications represents a valuable direction for future investigations.

Turbulence was modeled within a steady RANS framework. The same RANS k-ε closure was employed across the computational domain (bath, coil, and fire-tube) to ensure consistency of momentum and thermal transport predictions, and buoyancy effects were included through density variations in the body-force term. Near-wall treatment was implemented using wall functions, with meshes designed to maintain wall-adjacent resolution within a wall-function-appropriate y^+^ range. Given the strongly buoyant and stratified nature of the bath flow, model-form uncertainty is acknowledged; however, the primary circulation topology and thermal stratification trends were found to be qualitatively robust. More advanced transient turbulence treatments, such as URANS or LES, are therefore recommended for future studies.

The conclusion regarding the dominant influence of high excess air on overall heater efficiency should be interpreted at a qualitative and engineering level. While elevated excess air is known to increase sensible heat losses through the stack, the present model does not explicitly resolve flue-gas composition, stack O_2_/CO/CO_2_ balances, or detailed radiative heat transfer, nor does it rely on direct measurements of excess-air percentage. Validation was primarily performed using stack temperature data, which provides an indirect but practically relevant indicator of thermal losses. Consequently, the present results support a trend-based interpretation rather than a fully quantitative loss decomposition. A comprehensive quantitative assessment of excess-air-driven losses would require additional experimental data and radiation-resolved combustion modeling, which is recommended for future studies.

### Conclusions

This study presents a comprehensive 3D conjugate heat transfer CFD analysis of an industrial water-bath heater with integrated coil and fire-tube geometries, validated against field measurements. The results provide practical insights for operational optimization, including the influence of bath circulation patterns, coil duty distribution, and fire-tube thermal stratification. These findings highlight the novelty of our work in delivering both detailed thermal modeling and actionable recommendations for improving heater performance in real industrial settings.

*Practical implications.* Three levers emerge that can improve performance without altering the heater topology: (1) air-side tuning—modest reductions in excess air and/or adjusted primary/secondary splits (optionally mild air preheating) to curb stack losses while limiting wall hot spots; (2) bath-side management—gentle recirculation or simple baffles to weaken stratification and equalize coil exposure; and (3) coil optimization—pass placement/pitch to align the hottest bath zones with early passes and reduce approach length.

*Limitations and next steps.* The analysis assumed steady operation with methane as a fuel surrogate and RANS turbulence closures; pollutant kinetics and detailed radiation/soot coupling were outside the scope. Future work should extend the framework to transient start-up/shutdown, measured gas compositions and secondary-air distributions, and alternative firing strategies (e.g., blower-assisted burners), enabling a direct link between thermal performance, efficiency, and durability (corrosion/boiling risk) over the heater life cycle.

## Resource availability

### Lead contact

Further information and requests for resources should be directed to and will be fulfilled by the lead contact, Hamidreza Mortazavy Beni.

### Materials availability

This study did not generate new physical materials.

### Data and code availability

The datasets generated and analyzed during the current study are available from the corresponding author upon reasonable request. The CFD simulation files, processed datasets, and supporting numerical results are available upon request.

## Acknowledgments

The authors gratefully acknowledge the support of the operating team of the investigated natural gas city gate station for facilitating field measurements and providing access to operational data used for model validation. The authors also acknowledge the use of ChatGPT (OpenAI) for language editing and grammar review of selected sections of the manuscript. All scientific content, analyses, and conclusions remain the sole responsibility of the authors. Funding: This research received no external funding.

## Author contributions

H.M.B.: supervision, conceptualization, methodology, software, formal analysis, investigation, and writing – original draft; S.H.S.: validation, data curation, visualization, and writing – review and editing; A.A.: visualization, resources, and writing – review and editing; H.M.: conceptualization, methodology, software, formal analysis, investigation, and writing – original draft.

## Declaration of interests

The authors declare no competing interests.

## STAR★Methods

### Key resources table

**Table:** 

REAGENT or RESOURCE	SOURCE	IDENTIFIER
Water (bath fluid)	Current study	H_2_O
Methane (natural gas surrogate)	Current study	CH_4_
ANSYS Fluent	ANSYS Inc.	Version 2025 R1
ANSYS Meshing	ANSYS Inc.	Version 2025 R1
Standard k-ε turbulence model	ANSYS Fluent	RANS k-ε
Meteorological data	Public meteorological database	Weather dataset
CFD simulation datasets	Current study	Available upon request
